# Comparison of the Hydrodynamic Profile Between Competitive Triathletes and Swimmers

**DOI:** 10.3390/jfmk11010010

**Published:** 2025-12-26

**Authors:** Lawinya Assíria-Costa, Marta L. Machado, Catarina C. Santos, Mário J. Costa

**Affiliations:** 1 Centre for Sports Research, Education, Innovation and Intervention in Sport, CIFI2D, Faculty of Sport, University of Porto, 4200-450 Porto, Portugal; lawinyaamorimm@gmail.com (L.A.-C.); up202007622@edu.fade.up.pt (M.L.M.); 2 Department of Sport Sciences, Exercise and Health, University of Trás-os-Montes and Alto Douro, 5000-801 Vila Real, Portugal; catarinasantos@utad.pt; 3 Higher Education School, Polytechnic of Coimbra, 3045-093 Coimbra, Portugal; 4 Porto Biomechanics Laboratory, Faculty of Sport, University of Porto, 4200-450 Porto, Portugal

**Keywords:** triathlon, swimming, training, hydrodynamics, kinetics

## Abstract

**Objectives:** This study aimed to compare the hydrodynamic profile between triathletes and competitive swimmers and to establish associations with short- and middle-distance performance. **Methods:** A total of 18 male athletes, including 10 swimmers and 8 triathletes, all registered in their respective federations, underwent assessments of passive drag, active drag and power, tethered swimming force, kinematics, and performance over a 200 m and 25 m front crawl. Group comparisons were performed using either Student’s *t*-test or the Mann–Whitney U test at a significance level of *p* ≤ 0.05. **Results:** The triathletes presented higher passive drag and lower levels of force and power to overcome drag. Correlation analysis showed that, among the triathletes, both times at 200 m and 25 m were associated with mean passive drag (r = 0.68 to 0.86) and power (r = −0.58 to −0.80), whereas in swimmers, the mean in-water force was the single variable associated with time at 25 m (r = −0.51). **Conclusions:** There is a clear hydrodynamic superiority of swimmers compared to triathletes, reflecting their higher mean swimming velocity due to a greater ability to apply force. This suggests that specific technical interventions for triathletes, focusing on drag reduction and improvements in propulsive power, are needed to close this gap with swimmers.

## 1. Introduction

Triathlon is an endurance sport that requires athletes to perform beyond their aerobic capacity, directing attention to refined technical proficiency to optimize movement efficiency during prolonged efforts [1]. Given its complex structure, the training preparation must be approached from a biophysical perspective, integrating physiological and biomechanical variables that together determine the overall performance [2]. Although previous studies have predominantly focused on physiological responses to a single discipline [3], after a full race [4] or during transitions [5], there is limited research into biomechanical characteristics of triathletes.

Technical mastery should be seen as a decisive factor for triathlon performance. Here, proficiency is expected to directly contribute to reducing energy expenditure and sustaining performance across the different stages of the race [6]. In this context, biomechanics play a strategic role in identifying more economical movement patterns, thereby promoting more effective management of physical effort, especially in long-duration events [7]. This becomes particularly relevant in the swimming segment, where a poor technique, such as inadequate coordination or insufficient balance, increases hydrodynamic drag and reduces stroke efficiency [8]. These factors may not only compromise split times but also induce early fatigue that directly impairs subsequent cycling and running segments [9]. Therefore, technical mastery in swimming is essential not only to achieve a better split time but also to optimize energy expenditure, sustaining higher effort levels in the following phases of the race [10].

Recent reviews have shown that most studies on the swimming biomechanics of triathletes predominantly addressed kinematic variables such as stroke length and stroke rate, as well as the influence of wetsuits on stroke characteristics [2]. Indeed, a clear difference is observed in coordination or technical capacity when comparing triathletes with swimmers [11,12]. While these studies have greatly contributed to understanding triathletes’ technical adaptations, some specific hydrodynamic aspects remain underexplored. The hydrodynamic profile in swimming is one of the components from a deterministic model for performance that includes a set of variables like drag, propulsion, or power [13]. The understanding of passive or active drag and the force or power required to overcome drag are key aspects that regularly determine the competitive level of swimmers [14].

Studies examining the association between these hydrodynamic variables and triathletes’ performance under controlled conditions are scarce, limiting the practical applicability of available evidence for technical training interventions [15,16]. This literature gap highlights the need for experimental studies that allow for integrated hydrodynamic analysis. Testing swimmers is a perfect way to establish performance benchmarks, quantify the magnitude of the hydrodynamic gap, or identify specific deficits in triathletes. This will contribute to a more accurate characterization of triathletes’ hydrodynamic profiles and to an understanding of how far those are from swimmers.

The present study aimed to (i) compare the hydrodynamic profile between competitive swimmers and triathletes and (ii) examine the association between the hydrodynamic profile of both groups and maximal short- and middle-distance swimming performance. We hypothesized that (i) swimmers would demonstrate a more efficient hydrodynamic profile than triathletes; (ii) both swimmers and triathletes were expected to show associations between hydrodynamic variables and performance in short- and middle-distance swimming.

## 2. Materials and Methods

### 2.1. Participants

A cross-sectional comparative research design was used for this study. A convenience sample of 18 male participants was divided into two groups: 8 triathletes (35.85 ± 10.13 years) and 10 swimmers (21.48 ± 4.32 years). Inclusion criteria were defined as follows: (i) age ≥ 15 years; (ii) a minimum of three years of competitive experience within an integrated competition framework; (iii) active affiliation with the Portuguese Swimming Federation or the Portuguese Triathlon Federation during the 2024–2025 competitive season; and (iv) being healthy at the time of data collection, with no history of injuries or illnesses in the previous six months. All participants were informed about the study procedures and potential risks and signed an informed consent form. The study was conducted in accordance with the recommendations of the Declaration of Helsinki regarding research involving human participants.

### 2.2. Procedures

Athletes were assessed in two separate sessions in a 25 m swimming pool with water temperature at 27–28 °C. The first session was used for anthropometric characterization and in-water assessments of swimming kinematics, as well as passive and active drag measurements. The second session was used to apply an in-water force test and a 200 m front crawl trial. All athletes were familiarized with all the tests after a pilot study conducted few weeks before. They were dressed in the regular swimming shorts they used on a regular basis in their swimming training sessions. A standardized warm-up was performed in both sessions prior to in-water testing, consisting of 200 m of continuous swimming, followed by 4 × 50 m (25 m progressive and 25 m easy) and 100 m of recovery. Athletes were instructed to refrain from intense exercise prior to the testing sessions in the two days before and during data collection.

Anthropometric characterization was obtained according to previously established protocols [17], with athletes wearing standard swim attire (swimsuit and cap). Measurements included body mass (BM, kg) and stature (cm), recorded using a digital scale (TANITA, BC-730, TANITA Corporation, Tokyo, Japan) and a stadiometer (SECA, 242, SECA Gmbh & Co. kg, Hamburg, Germany), respectively. Upper limb length (ULL, cm) and arm span (AS, cm) were measured using a measuring tape (Rosscraft, Rosscraft Innovations Inc., Surrey, BC, Canada). The trunk transverse surface area (TSA, cm^2^) was calculated by digital photogrammetry using a photographic camera and a calibration object, as described by [18]. All images were exported to a screen-based software (Universal Desktop Ruler, v3.8, AVPSoft, Pittsburgh, PA, USA).

Active drag assessment was performed using the velocity perturbation method [17], which requires adding a resistance device while swimming (please see Figure 1 from [19]). Each athlete completed 2 × 25 m maximal-effort front crawl in randomized order, with and without a perturbation device attached. The test started with the athletes in the water, with no disturbance in adjacent lanes, and included a push-off from the wall while minimizing the underwater phase. Active drag (D*_a_*) was calculated using the velocity differential between the two repetitions according to the following equation:

(1) $$D_{a}=\frac{D_{b}\cdot v_{b}\;\cdot {\;v}^{2}}{v^{3}-v_{b}^{3}}$$

where D*_a_* is the active drag, D*_b_* is the resistance of the device provided by the manufacturer, *v*_b_ is the mean velocity with the device, and *v* is the mean velocity without the device. Subsequently, the active drag coefficient (CD*_a_*) was calculated using the following equation [19]:

(2) $$CD_{a}=\frac{2\cdot D_{a}}{\rho \cdot S\cdot v^{2}}$$

where ρ represents water density (1000 kg/m^3^), S is the swimmer’s frontal area estimated through photogrammetry, and *v* is the swimming velocity without the device. Finally, the power generated in water to overcome the drag force was calculated using the following equation:

(3) $$P=D_{a\;}\cdot v$$

where *P* represents hydrodynamic power (W), D*a* is active drag (N), and *v* corresponds to the mean swimming velocity without the resistance device (m·s^−1^).

The kinematic variables were obtained between the 13th and 24th meter of the pool length during the 25 m trial without the perturbation device. The analysis was performed by two expert investigators holding a PhD in Sports Sciences and former swimming coaches, who routinely conducted this type of measurement. After computing independent ICCs for all variables, a mean ICC = 0.96 was observed for inter-rater reliability. The mean value of the two assessments in all variables was used for further analysis. Swimming velocity (*v*, m·s^−1^) was calculated by dividing the distance by the time spent to cover the 25 m. Stroke frequency (SF, in cycles/min, later converted to Hz) was measured by the experts positioned in the lateral side of the pool using a 3-base stroke rate chronometer (Golfinho Sports MC 815, Aveiro, Portugal) by timing three consecutive upper-limb cycles. Stroke length (SL, in m) was determined by dividing *v* by SF, as described by Craig et al. [20]. Subsequently, the stroke index (SI, in m^2^·s^−1^) was obtained by multiplying *v* by SL [21].

To assess passive drag, each athlete was towed in a fundamental hydrodynamic position (i.e., streamline), which is characterized by keeping the head in a neutral position aligned with the spine, with the arms extended above the head and with the hands overlapped, while the trunk is kept straight and the legs are kept together and fully extended [22]. An electromechanical device (Swim-Spektro, Talamonti, Loreto Aprutino, Italy) was used to tow the athletes at two distinct velocities (1.0 and 1.5 m·s^−1^). The towing velocities were pre-determined previously in a pilot study, these being a range of velocities that both groups were able to attain while swimming. The maximum and mean passive drag were calculated from the force required to maintain constant velocity between the 10th and 20th meter.

The in-water force was assessed using a tethered swimming test. The athletes remained at a distance of 5 m from the wall, connected to an integrated force assessment system (Swim-Spektro, Talamonti, Italy) via a cable and a waist belt. The system was calibrated as previously described by Cortesi et al. [23]. To avoid inertial effects, the athletes began the test by swimming at a low intensity for 5 s, immediately followed by 30 s at maximal intensity, breathing ad libitum. One evaluator used an auditory signal to determine the start and end of the test. Data was recorded at a sampling rate of 100 Hz using the system’s proprietary software (DB:4, Talamonti Spa, Ascoli Piceno, Italy). Signal processing was subsequently performed using a MATLAB routine (v.0.5, R2023b, MathWorks, Inc., Natick, MA, USA), which included smoothing with a 10 Hz filter and correction for the 25° angle formed between the system and the water surface. This allowed for accurate determination of the horizontal component of maximal force (Fmax, N) and mean force (Fmean, N). Instantaneous force values from the first 2 s were discarded due to initial cable tension.

After ensuring full recovery, a 200 m front crawl time trial was performed (T200 m) to serve as the dependent variable and as an indicator of middle-distance performance, suitable for associating the hydrodynamic profile of both groups. The 25 m time (T25) was obtained from the velocity perturbation test, specifically from the trial performed without the perturbation device, and was considered as the short-distance performance variable. Both tests began with a push-off start with the swimmers alone and without any other swimmers in the nearby lanes. The swimmers were instructed to perform at their best on the day of data collection.

### 2.3. Statistical Procedures

Mean and standard deviation were used as measures of central tendency. Data normality was tested using the Shapiro–Wilk test. After the homogeneity of variance was checked, the group comparisons were performed using either Student’s *t*-test or the Mann–Whitney U test (independent samples). Effect size was calculated using Cohen’s *d* (95% CI), interpreted as trivial if *d* < 0.2, small if 0.2 ≤ *d* < 0.5, and large if *d* ≥ 0.5 [24]. The association between biomechanical parameters and performance was determined using Pearson’s or Spearman’s correlation, as appropriate, and interpreted as high if r ≥ 0.60, moderate if 0.30 ≤ r < 0.60, and low if r < 0.30 [25]. All statistical analyses were conducted using SPSS, version 27 (IBM, SPSS Inc., Chicago, IL, USA), with the significance level set at *p* ≤ 0.05. Graphical representations were created using GraphPad Prism (v.9, GraphPad Software, San Diego, CA, USA).

## 3. Results

Table 1 presents a comparison of anthropometric characterization between the competitive swimmers and triathletes, indicating that the swimmers had a larger arm span.

Table 2 presents a comparison of kinematic and hydrodynamic variables between the triathletes and swimmers. The *v*, SL, and SI were higher in the swimmers, with large effect sizes. The SF was the single kinematic variable that did not differ between groups, but a large effect was observed. Regarding the hydrodynamic variables, it is worth noting that Da should be interpreted in conjunction with power for better clarification. The swimmers showed higher values of Da, accompanied by greater Wd, but similar values of CDa compared to the triathletes.

**Figure 1:**
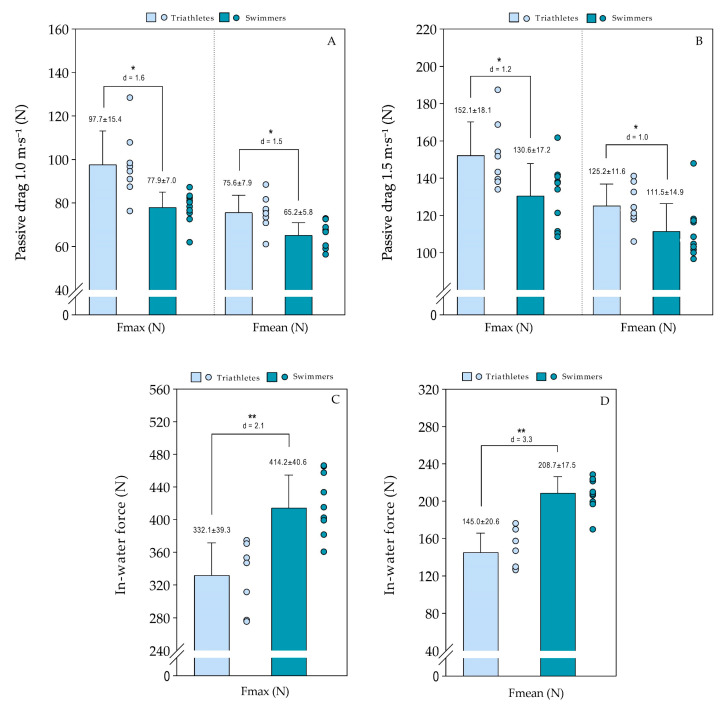
Comparison of passive drag (panels **A**,**B**) and in-water force (panels **C**,**D**) between triathletes (light blue) and swimmers (dark blue). * *p* ≤ 0.05, ** *p* ≤ 0.01.

Figure 1 illustrates the comparison of maximum and mean forces (drag and propulsion) between triathletes and swimmers. Triathletes showed higher passive drag than swimmers, with a large effect size, at both towing velocities. Swimmers demonstrated higher Fmax and Fmean values compared with triathletes, also indicating a large effect size.

**Figure 2:**
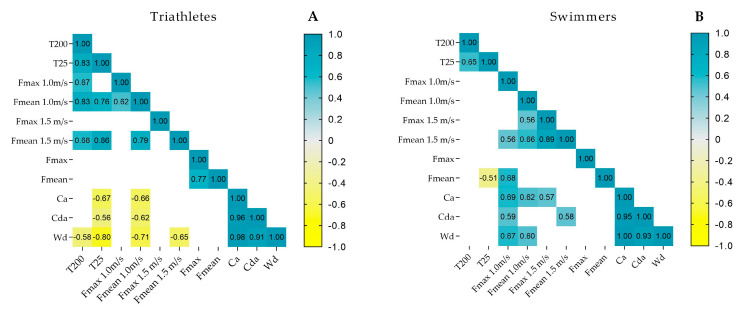
Association matrix between short- or middle-distance performance and hydrodynamic variables in triathletes (panel **A**) and swimmers (panel **B**).

Figure 2 presents the significant associations (*p* ≤ 0.05) between the hydrodynamic profile and T25 or T200 in both groups. The triathletes (panel A) showed a negative association between T25 and Wd (r = −0.80), Ca (r = −0.67) and, CDa (r = −0.62), while a positive association was observed for mean passive drag at 1.0 m·s^−1^ (r = 0.76) and 1.5 m·s^−1^ (r = 0.86), suggesting that lower power levels and higher drag values were associated with poorer performance. A similar trend was found in the T200, which was negatively associated with Wd (r = −0.58), while positive associations were found for mean and passive drag at 1.0 m·s^−1^ (r = 0.83) and 1.5 m·s^−1^ (r = 0.68). In the swimmers (panel B), a single negative association was observed between T25 and mean in-water force (r = −0.51).

## 4. Discussion

This study aimed to compare the hydrodynamic profile between competitive triathletes and swimmers and analyze the association between the hydrodynamic profile of both groups with the maximal short- and middle-distance performance.

The results regarding kinematic variables clearly demonstrated the technical superiority of swimmers compared with triathletes, with the swimmers showing higher values of *v*, SL, and SI. These findings are consistent with previous studies that attribute such differences to the greater water training volume and technical specialization that swimmers develop through their daily practice [26,27]. On the other hand, the absence of differences in SF suggests that efficiency is not associated with the constant repetition of the stroke, but rather with its amplitude and quality.

A surprising result was the higher Da observed in swimmers. Although the literature often associates lower Da values with greater efficiency [12], these results indicate that high Da values may coexist with high performance, because they are accompanied by greater Wd and better technique. This means that Da should not considered independently of the other variables. Furthermore, no differences were found in CDa between groups, suggesting that hydrodynamic efficiency, when normalized to the transverse surface area, may be similar in both triathletes and swimmers. While the hydrodynamic posture itself may be comparable, most differences arise primarily from force application and body alignment during active swimming. Therefore, the main differentiating factor appears to be the propulsive capacity, which enabled the swimmers to achieve higher velocities in the 25 m effort.

The analysis of the relationship between Wd and *v* revealed that triathletes exhibited lower Wd levels and *v* in comparison to swimmers. This suggests that performance is determined not only by the amount of power generated but also by how effectively that power can be translated to overcome drag and achieve higher velocities (i.e., swim faster). In fact, the observed dispersion between groups indicates that the efficiency of converting propulsive force into forward motion is a critical factor. Swimmers demonstrated a greater ability to transform generated energy into displacement, whereas triathletes appeared less effective at converting this effort into velocity, likely due to lower technical specialization in the propulsive stroke. Indeed, force application in water differs significantly when swimmers of different expertise levels are compared [28], which may similarly explain the differences observed between the swimmers and triathletes.

Regarding passive drag, triathletes presented higher mean and maximal values than swimmers at both towing velocities. This finding aligns with the results of Chatard and Wilson [29], who reported slightly higher passive drag in triathletes compared with swimmers, even though the two groups were not directly compared. The greater resistance to forward motion observed in triathletes may be attributed to differences in body position, particularly in maintaining the hydrodynamic characteristics. Hypothetically, the swimmers, having slimmer trunks and narrower transverse diameters, exhibited reduced passive drag during gliding, highlighting the strong influence of external body morphology on hydrodynamic resistance [30]. More expert swimmers also tend to display better body alignment and postural control (e.g., through head position, core and limb contraction), thereby reducing drag more effectively [22]. These findings are particularly important, as passive drag is often neglected in training programs. For triathletes, who spend less time in water, optimizing body position may represent a high-impact and low-cost strategy. Although wetsuits can sometimes mitigate these effects in competitions [31], the implementation of simple technical strategies, such as visual feedback and drills focused on alignment or active buoyancy exercises, may yield significant improvements in body position and swimming economy.

The tethered swimming tests revealed substantial and expected differences between groups, with swimmers showing higher maximal and mean force. Although previous studies have demonstrated greater swimming economy and lower energy cost in swimmers [10,32], such advantages have often been attributed to training volume. The force measured in tethered swimming reflects the potential to apply force in water but not necessarily its conversion into propulsive force. It could be argued that swimmers might also display higher force values in dry-land tasks involving similar movement patterns. Although this study did not assess such “force transfer,” the findings suggest that differences between groups also lie in the capacity to effectively apply force in the water. This indicates that triathletes, despite having a good force-generation capacity, often fail to effectively convert that force into forward motion, highlighting a technical limitation within the stroke cycle.

Overall, the combined analysis of kinematics, drag, and in-water force capacity highlights that performance differences between swimmers and triathletes arise from the interaction, rather than the isolated effects of these variables, which could be reasonably attributed to technical specialization over the years. The higher *v*, SL, and SI of swimmers reflect a more effective use of the propulsive phase, which not only amplifies stroke amplitude but also enables greater translation of force into forward motion. Although swimmers displayed higher active drag, this did not hinder their performance. Instead, it coexisted with better technique and higher drag force values, indicating that greater drag can be offset, or even leveraged, when accompanied by more efficient propulsive mechanics. The lack of differences in the drag coefficient further underscores that hydrodynamic efficiency per unit surface area is comparable between groups, shifting the focus toward the swimmers’ enhanced capacity to convert force into displacement.

The correlation analyses revealed distinct patterns between groups. In triathletes, variables such as passive drag, active drag, and power were associated with both T25 and T200 performance. This aligns with previous research, which identified hydrodynamics as the main determinant of performance in less specialized athletes [16]. In contrast, swimmers exhibited a single correlation, which may indicate a multifactorial performance model where physiological, technical, and other factors may interact to reach a better outcome. Therefore, while performance in swimmers depends on a complex set of variables [33], the most evident performance gains for triathletes lie in improving the hydrodynamic profile.

This underscores the need for targeted training programs focusing on the relationship between stroke technique and resistance to forward motion, thereby maximizing the return on training time dedicated to swimming in triathlons. Triathlon coaches should emphasize reducing passive drag through improved body alignment, streamlined breathing, and targeted technique drills, as triathletes show greater drag and lower propulsive power than swimmers. Training should also include resisted swimming and specific strength work to enhance force application, which would strongly influence their 25 m and 200 m performance.

Ultimately, some limitations could be acknowledged and surpassed in future studies: (i) the low sample size with an heterogeneous group of triathletes, particularly in terms of the age range, and their mean age in comparison to swimmers, which could lead to some variability in data interpretation; (ii) the hydrodynamic variables were estimated and not directly measured, which may be sensitive to variations arising from all-out efforts; (iii) the lack of direct physiological measurements, which prevented evaluating whether differences in power application were due purely to technique or also to conditioning; (iv) the use of 25 m and 200 m for performance distances, which may have been shorter than what the group of triathletes were used to swimming in competition and training, leading to some kind of underestimation of their real hydrodynamic capacity; and (iv) the use of specific towing velocities, rather than velocities based on a percentage of each participant’s maximum.

## 5. Conclusions

A clear hydrodynamic superiority was found in swimmers, reflected in a higher mean swimming velocity due to their greater ability to apply force. In addition, it was observed that, particularly among triathletes, hydrodynamic variables such as the power to overcome drag and both passive and active drag parameters were strongly associated with short- and middle-distance performance. These associations reinforce the relevance of targeting hydrodynamic characteristics in technical training interventions to optimize swimming performance, especially in triathletes.

## Figures and Tables

**Table 1:** Anthropometric characterization and comparison between triathletes and swimmers.

	Triathletes (Mean ± SD)	Swimmers (Mean ± SD)	*p*	*d*	95% CI
BM (kg)	71.99 ± 7.84	73.73 ± 5.17	0.578	0.86	−0.11, 1.83
Stature (cm)	177.65 ± 7.23	181.12 ± 5.68	0.270	0.53	−0.42, 1.48
AS (cm)	181.28 ± 8.78	191.53 ± 7.71	0.018	1.24	0.23, 2.25
ULL (cm)	82.69 ± 4.93	87.00 ± 5.30	0.096	0.88	−0.09, 1.85
TSA (cm^2^)	845.80 ± 143.50	863.39 ± 129.58	0.788	0.13	−0.8, 1.06

BM, body mass; AS, arm span; ULL, upper-limb length; TSA, trunk transverse surface area.

**Table 2:** Comparison of kinematic and hydrodynamic variables between triathletes and swimmers retrieved from the velocity perturbation method.

Variables	Triathletes (Mean ± SD)	Swimmers (Mean ± SD)	*p*	*d*	95% CI
***v*** (m·s^−1^)	1.38 ± 0.14	1.75 ± 0.07	0.009	3.49	1.97, 5.02
SF (Hz)	0.85 ± 0.12	0.92 ± 0.07	0.134	0.74	−0.22, 1.70
SL (m)	1.65 ± 0.17	1.92 ± 0.09	<0.001	2.02	0.86, 3.18
SI (m^2^·s^−1^)	2.28 ± 0.34	3.35 ± 0.16	<0.001	4.20	2.47, 5.93
D_a_ (N)	31.06 ± 10.37	51.80 ± 22.55	0.029	1.14	0.13, 2.15
CD_a_ (dimensionless)	0.38 ± 0.09	0.39 ± 0.17	0.903	0.07	−0.86, 1.00
Wd (W)	43.66 ± 17.41	91.02 ± 40.69	0.007	1.44	0.39, 2.59

*v*, mean swimming velocity; SF, stroke frequency; SL, stroke length; SI, stroke index; D_a_, active drag; CD_a_, active drag coefficient; Wd, power to overcome drag; *d*, Cohen’s effect size.

## Data Availability

The authors confirm that the data supporting the findings of this study are available within this article.
